# Updating Insights into the Regulatory Mechanisms of Calcineurin-Activated Transcription Factor Crz1 in Pathogenic Fungi

**DOI:** 10.3390/jof8101082

**Published:** 2022-10-14

**Authors:** Yangyang Yang, Pengdong Xie, Yongcai Li, Yang Bi, Dov B. Prusky

**Affiliations:** 1 College of Food Science and Engineering, Gansu Agricultural University, Lanzhou 730070, China; 2 Department of Postharvest Science, Agricultural Research Organization, Volcani Center, Rishon LeZion 7505101, Israel

**Keywords:** calcium homeostasis, Crz1, fungi, cellular functions, molecular regulatory mechanisms, cross-talk

## Abstract

Ca^2+^, as a second messenger in cells, enables organisms to adapt to different environmental stresses by rapidly sensing and responding to external stimuli. In recent years, the Ca^2+^ mediated calcium signaling pathway has been studied systematically in various mammals and fungi, indicating that the pathway is conserved among organisms. The pathway consists mainly of complex Ca^2+^ channel proteins, calcium pumps, Ca^2+^ transporters and many related proteins. Crz1, a transcription factor downstream of the calcium signaling pathway, participates in regulating cell survival, ion homeostasis, infection structure development, cell wall integrity and virulence. This review briefly summarizes the Ca^2+^ mediated calcium signaling pathway and regulatory roles in plant pathogenic fungi. Based on discussing the structure and localization of transcription factor Crz1, we focus on the regulatory role of Crz1 on growth and development, stress response, pathogenicity of pathogenic fungi and its regulatory mechanisms. Furthermore, we explore the cross-talk between Crz1 and other signaling pathways. Combined with the important role and pathogenic mechanism of Crz1 in fungi, the new strategies in which Crz1 may be used as a target to explore disease control in practice are also discussed.

## 1. Introduction

Ca^2+^, as a second messenger, plays an important role in the regulation of biological function in cells. Unlike other second messengers, Ca^2+^ does not need to be synthesized but instead controls intracellular Ca^2+^ content through a series of complex regulatory mechanisms when responding to external signals. The CaN-Crz1 signaling cascade in fungal cells can be activated by different external stimuli, such as high temperature, low temperature, hypertonicity, alkalinity, oxidative stress, ethanol stress, light sources, antifungal drugs and others. The signal transduction mediated by Ca^2+^ can cause an instantaneous increase in intracellular Ca^2+^, which is generally considered to be the switch to turn on the signaling pathway [1,2,3,4,5,6,7]. The transient increase of intracellular Ca^2+^ content is caused by the entry of extracellular Ca^2+^ into cells through Ca^2+^ channel proteins Mid1 and Cch1 on the plasma membrane, or the release of Ca^2+^ from the intracellular calcium pool [8,9,10]. Intracellular free Ca^2+^ combines with calmodulin (CaM) to form a Ca^2+^/CaM complex and then activates calcineurin (CaN), which further dephosphorylates transcription factor Crz1 and allows it into the nucleus to regulate the expression of target genes [11]. The pathway is considered the Ca^2+^/calmodulin/Crz1 signaling pathway, also known as the CCS (calcium cell survival) pathway [8]. At present, the calcium signaling pathway has been systematically studied in mammals, parasites and yeasts [12,13,14,15,16,17,18]. Various components of the calcium signaling pathway play an important role in vascular development, axon outgrowth, stress response and glycogen synthesis in organisms [19,20,21,22,23,24]. This review briefly summarizes the calcium channels, calcium pumps and Ca^2+^ sensor proteins of the calcium pathway system in fungi, pointing out that the calcium homeostasis system is involved in a variety of life processes, such as cell growth, conidia production, stress response and maintenance of normal organelle function. We highlight recent findings on how transcription factor Crz1 regulates growth and development, stress responses, pathogenicity of pathogenic fungi and its regulatory mechanisms based on discussing the structure and localization of Crz1. In addition, cross-talk between Crz1 and other signaling pathways and how recent advances in our understanding of CaN-Crz1 signaling cascade might be used in practice to explore new strategies for disease control are also discussed.

## 2. Calcium Signaling Pathway in Fungal Cell

**Figure 1:**
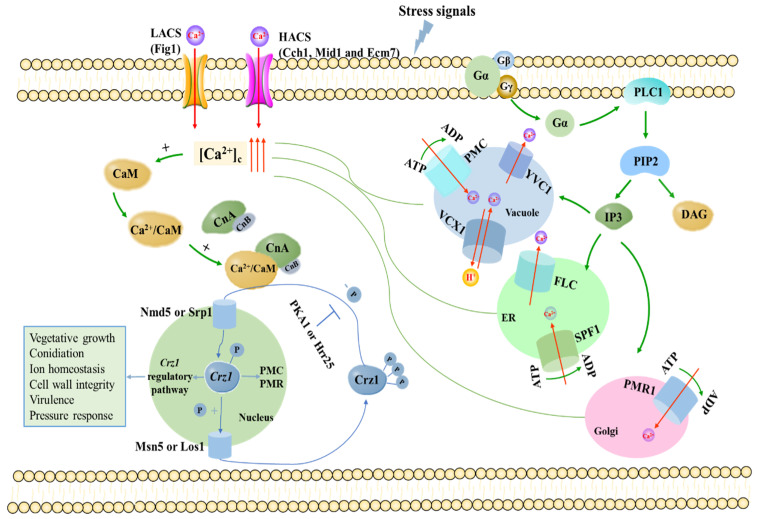
Pattern of calcium homeostasis system in fungi. When the cytosolic Ca^2+^ concentration increases, calmodulin activates calcineurin, which in turn dephosphorylates Crz1. Crz1 is then imported into the nucleus and induces or represses expression of its target genes. HACS: high-affinity calcium system; LACS: low-affinity calcium system; [Ca^2+^]_c_: cytosolic calcium concentration; CaM: calmodulin; CnA: calcineurin catalytic subunit; CnB: calcineurin regulatory subunit; Crz1: calcineurin responsive transcription; PMC: plasma membrane Ca^2+^-ATPase; PMR: plasma membrane ATPase-related pump; ER: endoplasmic reticulum; FLC: flavin carriers; PLC1: phospholipase C; PIP2: inositol-4,5-diphosphate; IP3: inositol triphosphate; DAG: diacylglycerol.

The calcium signaling system plays a very important regulatory role in the whole process of fungal growth and development. Imbalance in the calcium signaling system leads to abnormality of fungal cells in various aspects such as reproductive development, polar growth, cell differentiation and division, stress response and programmed death. Therefore, maintaining the stability of intracellular calcium levels is crucial for cell survival. Under normal physiological conditions, the concentration of cytoplasmic Ca^2+^ in fungal cells is in the low range of 50 to 100 nM [9,25]. The stability of Ca^2+^ levels in cells is controlled by a complex Ca^2+^ homeostasis regulatory system (Figure 1), which includes multiple Ca^2+^ channel proteins and pumps, as well as Ca^2+^ transporters, and many related proteins and enzymes in eukaryotes [9,25,26]. These components, mainly located on the plasma membrane or different subcellular organelles, are responsible for absorbing Ca^2+^ release from extracellular and intracellular calcium pools, thereby synergistically regulating the stability of Ca^2+^ levels in the cytoplasm and various organelles [27,28,29].

Two pathways have been reported to participate in extracellular Ca^2+^ uptake in fungi: the high-affinity Ca^2+^ transport system (HACS) and low-affinity Ca^2+^ transport system (LACS). The HACS, composed of Mid1 and Cch1, is responsible for Ca^2+^ uptake at low calcium concentrations (about 100 nM) [4,30,31]. Recently, Ecm7, a member of the PMP-22/EMP/MP20/Claudin superfamily of transmembrane proteins that includes γ-subunits of voltage-gated calcium channels, was identified as another subunit of HACS [32,33]. Cch1, the first Ca^2+^-related protein in the Ca^2+^/calmodulin/calcineurin/Crz1 signaling pathway, plays a critical role in regulating a variety of physiological activities activated by the calcium signaling system in fungal cells [34,35,36,37,38,39]. Mid1 and Cch1 are subject to feedback inhibition by calcineurin in a high calcium environment; then, the LACS plays a major role. The only known component of LACS to date is the membrane protein in Figure 1 [25,30,40]. The deletion of Figure 1 in fungi affects a wide range of cellular processes, such as sexual reproduction, mycelial growth, virulence and conidia production [41,42,43,44]. Recently, transient receptor potential (TRP) channels were found among mammals, flies, worms, ciliates, Chlamydomonas and yeasts [45]. The TRP channels act as sensors for various stresses, including temperature, pH, osmolarity and nutrient availability [46,47,48,49]. The first calcium-permeable TRP, initially isolated from *Arabidopsis thaliana*, can be activated by hyperosmotic shock and, therefore, was named calcium-permeable stress-gated cation channel 1 (CSC1) [47], which includes the PenV protein of *P. chrysogenum* and CefP of *A. chrysogenum* [50]. The Yvc1 channel protein located on the tonoplast is a homologue of mammalian transient receptor potential (TRP) channel protein responsible for the release of Ca^2+^ from the vacuole into the cytoplasm [10,51,52]. FLC was recently proposed as a member of the FLC family required for importing FAD into the endoplasmic reticulum, and it represent a conserved fungal gene family of integral membrane protein, spanning a TRP-like domain [49,53]. Some studies suggest FLC could act as either a calcium sensor or directly as a calcium channel [49].

There are many kinds of calcium pools in fungal cells, such as endoplasmic reticulum, Golgi apparatus and vacuoles. Different calcium pumps are distributed in these calcium pools, and are responsible for transporting Ca^2+^ from the cytoplasm to various organelles against the concentration gradient. For fungal cells, vacuoles rather than endoplasmic reticulum are the most important calcium pools, where the concentration of Ca^2+^ is about 10^4^ times that of cytoplasmic [54,55]. This large amount of Ca^2+^ storage is maintained by the action of two transporter proteins, Ca^2+^-ATPase Pmc1 and Ca^2+^/H^+^ exchanger Vcx1 [10,26,56,57,58,59,60]. Vcx1 belongs to the CAX superfamily of calcium-permeable ion exchangers [61,62,63]. When there is a burst in the cytoplasmic content of calcium, the Vcx1 transporter sequesters the calcium into the vacuoles. In addition to calcium, the Vcx1 protein transports Mn^2+^ ions, thus allowing *S. cerevisiae* to grow in high concentrations of either calcium or manganese ions [64]. Pmr1 (Plasma membrane ATPase related) is the first member of the secretory pathway Ca^2+^-ATPase (SPCA) family, which mediates the transport of Ca^2+^ and Mn^2+^ in Golgi under normal physiological conditions [56,65,66,67,68,69].

In order to precisely regulate intracellular calcium signals, organisms have also evolved several calcium-sensing proteins to respond to different ranges of Ca^2+^ concentration levels [70]. CaM, located downstream of phospholipase C [71] in the calcium signaling pathway, is a very important Ca^2+^ sensor that can sense the change of intracellular Ca^2+^ concentration and regulate a series of downstream target proteins by binding with Ca^2+^ [72,73,74]. CaN, as a Ca^2+^ and CaM dependent serine/threonine protein phosphatase, is composed of the catalytic subunit CNA and the regulatory subunit CNB [75,76,77,78,79], and is the central mediator of the Ca^2+^/calmodulin/calcineurin/Crz1 signaling pathway. In fact, calcineurin regulates the activity of diverse calcium transporters on the plasma membrane and is mainly responsible for calcium homeostasis [80]. Upon Ca^2+^ presence, the activated CaM binds to the CNA and CNB complexes to form a fully activated trimer [81], and then activated CaN dephosphorylates Crz1 and transfers it to the nucleus to regulate the expression of Crz1-dependent genes [11,82]. In fungi, the CaN-Crz1 signaling pathway is also conserved and involved in many biological processes, such as cell growth, infection structure differentiation, cell wall integrity, pathogenicity and stress response [83,84,85,86,87] (Figure 1). The Figure 1 only depicts genes in the calcium signaling pathway that are closely linked to Crz1 or directly regulated by Crz1.

## 3. Calcineurin-Activated Transcription Factor Crz1

### 3.1. Structure and Localization of Calcineurin Responsive Transcription Factor Crz1

Crz1 is the earliest identified downstream target protein of CaN in the Ca^2+^/CaM-CaN signaling cascade reaction [88]. Crz1 contains six important domains, including the C2H2 zinc finger DNA-binding domain, the calcineurin-docking domain (CDD), the serine-rich region (SRR), the nuclear export signal (NES), nuclear localization signal (NLS), and calcineurin docking motif (docking site to calcineurin, PIISIQ) [11,89,90,91,92,93,94]. The C2H2 zinc finger domain can bind to some target gene promoter response elements, which are called CDREs (calcineurin-dependent response elements). Therefore, a gene promoter sequence with this element is likely to be regulated by Crz1 [88]. The docking motif PIISIQ reported in *Saccharomyces cerevisiae* is the site of interaction between CaN and Crz1 [92]. The SRR structural domain, a serine-rich region containing several serine residues, is the target site for dephosphorylation of Crz1 by calcineurin and determines the localization and phosphorylation level of Crz1 [82,95]. Without external stresses or stimulus, Crz1 is localized in the cytoplasm, while upon increased Ca^2+^ concentration, CaN is activated to dephosphorylate Crz1, and then dephosphorylated Crz1 relocates to the nucleus for regulating targeted genes. This localization can be reversed by inhibitors, such as cyclosporine A, which inhibits CaN activity and redistributes Crz1 to the cytoplasm [11]. In addition, Crz1 can be phosphorylated in the presence of protein phosphokinase. The homologous protein Hrr25 of casein kinase 1 in mammals was detected by the high-throughput protein chip method [96]. In *S. cerevisiae*, Hrr25 plays a role in responding to DNA damage, mitosis and vacuole transport. In vivo, Hrr25 can bind to Crz1 and phosphorylate it to change its localization. The phosphorylated Crz1 is transported to the cytoplasm to avoid its accumulation in the nucleus. The ability of Crz1 to transport between cytoplasm and nucleus is regulated by NLS and NES. NLS and NES are able to form complexes with cellular input or output proteins, respectively. There is an NLS at the C-terminus of dephosphorylated Crz1, which can bind to the nuclear input protein Nmd5. Therefore, Nmd5 is responsible for transporting Crz1 to the nucleus. Interaction between NES and nuclear export protein Msn5 is responsible for nuclear export of phosphorylated Crz1 [73,82,97]. However, different Crz1 nuclear input and output proteins have recently been found in the industrial fungus *Penicillium oxalicum* [98]. Using tandem affinity purification combined with mass spectrometry (TAP-MS), no *Msn5* homologue was found in *P. oxalicum* instead of the nuclear transporter Los1. Therefore, it is more likely that PoCrz1 is exported from the nucleus through Los1 than through Msn5. Los1 and Msn5 play some overlapping roles in nuclear output [99]. In addition, *PoCrz1* enters the nucleus through Srp1 rather than Nmd5 [98]. These findings suggest that Crz1 transportation between cytoplasm and nucleus is also finely regulated.

### 3.2. Transcription Factor Crz1 Regulates Fungal Growth and Development

The transcription factor Crz1 regulates target genes and proteins through the calcium signaling cascade pathway and ultimately affects fungal growth, development and pathogenicity. Deletion of *Crz1* resulted in abnormal development of vegetative growth of most pathogenic fungi. The *ΔBcCrz1* mutant in *Botrytis cinerea* demonstrated impaired mycelial growth and abnormal branching on CM medium [90]. Similarly, the vegetative growth of *ΔFgCrz1* in *Fusarium graminearum* and *ΔAnCrz1* in *Aspergillus nidulans* shows severe defects [100,101]. However, in *Penicillium digitatum*, *Aspergillus fumigatus*, *Magnaporthe grisea* and *Verticillium dahliae*, the absence of *Crz1* has no significant effect on their vegetative growth [91,102,103,104]. In human pathogenic fungus *Candida lusitaniae*, the deletion of *Crz1* is associated with the loss of the ability to transform from yeast to hyphal morphology [105]. The cell structure of the WT and *Crz1* mutants in *Candida glabrata* was observed via transmission electron microscope and it was found that compared with WT, the *Crz1* mutants demonstrated irregular plasma membrane structure and abnormal organelles [106]. Formation and development of fungal conidia require Crz1. For example, after knocking out *Crz1* in *B. cinerea*, the *ΔBcCrz1* cannot produce sporophores or conidia [90]. The *ΔFgCrz1* in *F. graminearum* was unable to form perithecium, which affected its sexual development [100]. After the deletion of *Crz1*, *Valsa pyri* could not form a fruiting body structure [107]. Other studies have reported that the *A. nidulans* could open the calcium channel through the pressure sensor on the cell wall, and the CNA/Crz1 complex was activated, thereby promoting the polar growth of mycelia [108]. In a word, Crz1 is involved in various physiological functions of fungi, which we summarized in Table 1.

### 3.3. Transcription Factor Crz1 Is Essential for Fungal Pathogenicity

The virulence regulated by Crz1 was first studied in *C. albicans* [94,109], a human pathogenic fungus, and it was confirmed that the deletion of *Crz1* would reduce the virulence. Crz1 is also associated with the virulence of other *Candida* species. In emerging fungal pathogens *C. lusitaniae* and *C. glabrata*, it has been shown that the signal transduction pathway of CaN-Crz1 can control the virulence of the systemic infection model in mice [105,106,110]. Interestingly, the effect of Crz1 on virulence was also related to the specific niche of the host. For example, Crz1 is particularly important for murine eye infection, but it does not play a role in the murine urinary tract infection model [106]. It is well known that Crz1 is also necessary for mycelial growth, morphological transformation and spore and appressorium formation of filamentous fungi [89,90,91,102,103,104], on top of being the precursor for the formation and maintenance of pathogenicity of pathogenic fungi. In *Magnaporthe oryzae*, compared with the WT, the reduced pathogenicity of the *Crz1* knockout strain is mainly due to the decreased swelling pressure of appressorium, which leads to osmotic damage [89]. The reduction of appressorium swelling found in *ΔMgCrz1* is reported to be caused by disruption of lipid metabolism [103]. In *B. cinerea*, the absence of *Crz1* can cause defects in cell wall and membrane integrity, thus weakening the ability of hyphae to penetrate plant tissues [90]. The significantly decreased pathogenicity of the *ΔFgCrz1* in *F. graminearum* was suggested to be associated with impaired toxin DON biosynthesis [100]. In summary, through these studies on the pathogenic infection mechanisms of fungi pathogens, it was found that although Crz1 played a conservative role in fungi virulence or pathogenicity, the pathogenic mechanisms were different.

### 3.4. Transcription Factor Crz1 Involved in Fungal Stresses Responses

Fungi are frequently exposed to a variety of environmental stresses, including metal ions, oxidative stress, pH and cell wall interference agents. In order to cope with these environmental stresses, fungi evolve various strategies to quickly sense these signals, and then reduce the damage caused by environmental stresses. The transcription factor Crz1 is activated by stress-induced elevated Ca^2+^ levels and regulates the expression of related genes in response to these stresses. Crz1 is involved in the response of fungi to various stresses, as shown in Table 2.

#### 3.4.1. Transcription Factor Crz1 in Ion Stress Response

In fungi, the *Crz1* mutant is sensitive to ion stress, especially hypersensitivity to Ca^2+^, which has been reported in several studies [89,90,91,102,103,111,112] and may be due to the dephosphorylated Crz1 being transferred into the nucleus to induce the expression of multiple genes related to calcium ion stress, such as PMC and PMR [69,93,113,114]. However, sensitivity to other cation ions stresses such as Na^+^, Li^+^, Mg^2+^ and Mn^2+^ varies among *Crz1* deleted fungal species. In *A. fumigatus*, the *ΔAfCrz1* demonstrated strong sensitivity to Mn^2+^, but low sensitivity to Na^+^ and Li^+^ [91]. For *M. grisea*, the *ΔMgCrz1* was insensitive to Na^+^, Li^+^ and Mn^2+^ [89,103]. On the contrary, the *ΔBcCrz1* mutant demonstrated a strong sensitivity to these four ion stresses. In addition, it was found that the addition of Mg^2+^ restored growth defects and cell wall integrity in the *ΔBcCrz1* of *B. cinerea* [90]. These data suggest that ion stress responses and ion homeostasis regulated by Crz1 are a common feature in fungi, although there was species specificity.

#### 3.4.2. Transcription Factor Crz1 in Oxidative Stress Response

Yeast glutathione peroxidase GPX2 is a part of the antioxidant system that protects cells from oxidative stress. The expression of GPX2 induced by H_2_O_2_ is strictly regulated by transcription factor YAP1 and response regulator SKN7 [115,116]. Meanwhile, SKN7 has been found to be a multicopy enhancer of CaN-Crz1 dependent transcription in yeast, and SKN7 regulates calcineurin signaling by stabilizing Crz1 through direct protein–protein interaction [117]. The sensitivity of Crz1 to oxidative stress was also confirmed in *B. cinerea* [90], *M. acridum* [118] and *P. digitatum* [102]. The specific regulatory role of Crz1 in fungal pathogen response to oxidative stress needs to be further elucidated.

#### 3.4.3. Transcription Factor Crz1 in pH Stress Response

Crz1 is essential for tolerance to high pH conditions in yeast. Upon stimulation of alkaline conditions, Ca^2+^ enters the cytoplasm through the Cch1-Mid1 channel and then activates CaN to dephosphorylate Crz1 into the nucleus to induce several alkaline pH-responsive gene expressions, including *ENA1*, *PHO84*, *PHO89* and *PHO12* [119,120]. The colony growth rate of *ΔBcCrz1* slowed down under extreme pH (3 or 9). Interestingly, exogenous Mg^2+^ addition could restore the growth phenotype at pH 9, but the *ΔBcCrz1* growth defect phenotype did not recover at pH 3 [90].

#### 3.4.4. Transcription Factor Crz1 in Cell Wall Interference Agents

The growth of *Crz1* mutants in *P. digitatum*, *M. oryzae* and *B. cinerea* were seriously damaged in the medium containing cell wall inhibitors [89,90,102]. However, compared with the WT, the mycelial growth of *ΔVpCrz1* was significantly increased on CM agar medium containing SDS, CR or CFW, which was inconsistent with previous reports. It was suggested that *VpCrz1* acted as a negative regulator of cell wall stress in *V. pyri* [107]. Similarly, the *Crz1* mutant demonstrated resistance to SDS in human pathogenic fungus *Candida lusitaniae*, indicating that Crz1 negatively regulated cell membrane integrity, while Crz1 was found to respond to SDS by an unknown mechanism independent of CaN [105]. 

In addition, the involvement of Crz1 in fungal stress resistance was also reflected in the tolerance of antifungal drugs, temperature and ethanol. It has been reported that the damage of Crz1 in *S. cerevisiae* increases its sensitivity to azole drugs, while its overexpression reduces the sensitivity [7]. Similarly, Crz1 is responsible for azole resistance in *P. digitorum* as well as *ΔPdCrz1* reduced imidazole and difenoconazole tolerance [102]. In *C. neoformans*, Crz1 homologous phospholipid binding protein Cts1 was identified as a CaN substrate for high-temperature stress [121]. The *ΔCgCrz1* in *C. glabrata* could not grow as normally as the WT at 40 °C [106]. Ethanol was a common stress source in yeast. The cells lacking *Crz1* demonstrated poor adaptation to ethanol stress, while the multi-copy plasmid of Crz1 improved the tolerance to ethanol stress. Therefore, Crz1 was crucial for the survival of yeast cells under ethanol-induced stress [122]. It has been demonstrated in *C. neoformans* that Crz1 is involved in cell survival, biofilm formation and fluconazole sensitivity in the hypoxic environment [123].

**Table 1:** Regulatory roles of transcription factor Crz1 in fungi.

Fungal Species	Cellular Functions of Crz1	Selected References
*Alternaria alternata*	Infection structure differentiation Pathogenicity Vegetative growth Stress tolerance Cell wall integrity Melanin production Calcium homeostasis	[86]
*Magnaporthe oryzae*	Conidiation Ionic homeostasis Cell wall integrity Virulence	[89]
*Botrytis cinerea*	Vegetative growth Mycelial morphology Conidiation Cell wall integrity Virulence	[90]
*Fusarium graminearum*	Vegetative growth Sexual development Toxin synthesis Stress responses Virulence	[100]
*Penicillium digitatum*	Conidiation Virulence DMI resistance	[102]
*Magnaporthe grisea*	Conidiation Appressorium formation Calcium tolerance Melanin production Lipid metabolism Virulence	[103]
*Verticillium dahliae*	Microsclerotia development Melanin accumulation Cell wall integrity Virulence	[104]
*Candida lusitaniae*	Cell wall integrity ER stress Pseudohyphal growth Ca^2+^ homeostasis Virulence	[105]
*Candida glabrata*	Thermotolerance cell morphology Virulence ER stress tolerance	[106]
*Valsa pyri*	Fruiting body formation Mycelial morphology Virulence Cell wall perturbing agents resistance	[107]
*Cryptococcus neoformans*	Hypoxic adaptation Inbiofilm formation Cell wall integrity Fluconazole tolerance	[123]

**Table 2:** Stress responses regulated by transcription factor Crz1 in fungi.

Environmental Stresses	Fungal Species	Selected References
Ion stress	*Magnaporthe oryzae*	[89]
	*Botrytis cinerea*	[90]
	*Aspergillus fumigatus*	[91]
	*Penicillium digitatum*	[102]
	*Magnaporthe grisea*	[103]
	*Torulaspora delbrueckii*	[111]
	*Aspergillus nidulans*	[112]
Oxidative stress	*Botrytis cinerea*	[90]
	*Penicillium digitatum*	[102]
	*Saccharomyces cerevisiae*	[115,116]
	*Metarhizium acridum*	[118]
Alkaline stress	*Botrytis cinerea*	[90]
	*Saccharomyces cerevisiae*	[119,120]
Cell-wall-perturbing agents	*Magnaporthe oryzae*	[89]
	*Botrytis cinerea*	[101]
	*Penicillium digitatum*	[102]
	*Candida lusitaniae*	[105]
Antifungal agents	*Saccharomyces cerevisiae*	[7]
	*Penicillium digitatum*	[102]
High temperature stress	*Candida glabrata*	[106]
	*Cryptococcus neoformans*	[121]
Ethanol stress	*Saccharomyces cerevisiae*	[122]
Hypoxic stress	*Cryptococcus neoformans*	[123]

### 3.5. Molecular Regulatory Mechanisms of Transcription Factor Crz1 in Pathogenic Fungi

The zinc finger domain of Crz1 specifically binds to the 24 bp CDREs sequence to initiate target gene expression [88,124]. In *S. cerevisiae*, the core consensus site for Crz1 binding is 5′-GNGGCKCA-3′ [93], and the putative DNA common sequence bound by Crz1 in *Trichoderma reesei* was identified as 5′-GDGGCKBNB-3′ [125]. Therefore, we hypothesize that 5′-GNGGCK-3′ is a common sequence of Crz1-binding DNA. The target genes involved in ion homeostasis, cell wall maintenance, lipid synthesis, protein degradation and glucose metabolism are regulated by Crz1. Several studies have identified species-specific genes regulated by Crz1, and Crz1 can also be used as an inducer or inhibitor of gene expression. Crz1 is necessary for PMC and PMR to respond to Ca^2+^. PMC and PMR belong to the P-type ATPase superfamily, which can obtain energy by hydrolyzing ATP to drive Ca^2+^ transport from the cytoplasm to the vacuole and the Golgi, respectively, to maintain intracellular calcium homeostasis [67,113]. In fungi, the expression of *PMC* and *PMR* genes is significantly induced in response to Ca^2+^, but the expression levels are not highly activated in the *Crz1* mutants [89,91,102]. The reduced expression of these ATPases prevented the normal translocation of excess Ca^2+^ from the cytoplasm to various organelles, resulting in a disruption of calcium homeostasis, which may account for the sensitivity of *Crz1* mutants to Ca^2+^. *ENA1*, *ENA2*, and *ENA3* belong to the encoding plasma membrane Na^+^/Li^+^-ATPase, which are necessary for yeast survival under high Na^+^ and Li^+^ concentrations, and their expression is also induced by CaN in a Crz1-dependent manner [93,126]. In addition, other genes involved in ion homeostases such as *MEP1*, *ENB1*, *PHO84*, *PHO89* and *KHA1* are also regulated by CaN-Crz1 pathway [93]. Under external stress stimulation, the *β*-1,3 glucan synthase (FKS) and the chitin synthase (CHS) are essential for maintaining cell wall integrity. In the *Crz1* mutant, both *FKS* and *CHS* expression are disrupted [88,90,92,112]. Other genes involved in maintaining cell wall integrity such as *CRH1*, *RHO1*, *SCW10* and *KRE6* are also regulated by the CaN-Crz1 pathway [93]. In *P. oxalicum*, an industrial fungus, Crz1 plays a role in cellulase synthesis by regulating the expression of cellulose decomposition genes such as *cbh1*, *eg1* and *eg2* [98]. Expression of genes related to lipid and sterol metabolism such as *SUR1*, *CSG2*, *YSR3*, *ERG26*, *HES1* and *PLB3*, as well as genes involved in vesicular transport such as *GYP7*, *YPT53*, *YIP3*, *PEP12*, *RVS161*, *SHE4*, *CVT17*, *CVT19* and *VPS36*, all of which are regulated by Crz1, thus enables cells to maintain normal membrane function and complete the process of substance delivery to the cell surface [93]. However, studies have found that not all Crz1 functions depend on CaN. As demonstrated in *C. neoformans*, Crz1 exhibits a specific CaN-independent response to different environmental stress stimuli [127,128], Furthermore, in *C. dubliniensis*, Crz1 regulates haptotropic (surface-sensing) responses independently of CaN [129].

### 3.6. Cross-Talk between Transcription Factor Crz1 and Other Signaling Pathways

At present, it has been found that Crz1, a downstream transcription factor of the calcium signaling pathway, is not only related to calcium signaling but also participates in the transcriptional regulation of other signaling pathways. The cell wall integrity (CWI) pathway, one of the MAPK cascades pathways, maintains cell wall integrity by mediating cell wall biosynthesis. Since cell wall integrity is critical for cells to cope with environmental stress, CWI pathways need to cross-talk with other proteins or pathways to enhance their transduction ability [130,131]. Numerous studies have found that Crz1 maintains cell wall integrity by regulating genes involved in *CHS* and *FKS* biosynthesis [88,90,92,112,132,133]. Therefore, it is inferred that Crz1 cooperates with the CWI pathway to regulate cell wall integrity.

The high-osmolarity glycerol (HOG) pathway is used to regulate various stress genes for osmotic protection, and activation of this pathway is regulated by two upstream branches, one mediated by the *Sho1* sensor and the other by a system consisting of *Sln1*, *Ypd1* and *Ssk1* [134,135,136,137,138]. At the same time, Crz1 participates in the regulation of ion osmotic homeostasis by mediating the expression of ion transport genes [89,91,92,93,102,116]. Shitamukai et al. [139] found that there was a crosstalk relationship between the HOG and the CaN-Crz1 signaling pathway, and proved that there was an antagonistic effect between them. The CaN-Crz1 signaling pathway is involved in the downregulation of the HOG pathway by regulating the *Sln1* branch. In addition, the cyclic adenosine monophosphate-protein kinase A (cAMP-PKA) pathway is also antagonistic to the CaN-Crz1 signaling pathway. It was found that Crz1 is a substrate for PKA, which is functionally opposite to the CaN signaling pathway, and PKA can directly phosphorylate Crz1 to inhibit its nuclear localization and activity [140].

In *S. cerevisiae*, *Neurospora crassa* and mammals, it has been shown that external signals are sensed by G protein-coupled receptors (GPCRs) [141,142]. After sensing the stimulation of external signals, membrane binding receptors trigger G protein to dissociate Gα subunit from Gβ/γ subunit. The released Gα subunit activates phospholipase C (PLC), which hydrolyzes inositol-4,5-diphosphate (PIP2) to generate two important messenger molecules, diacylglycerol (DAG) and inositol-1,4,5-triphosphate (IP3) [143]. Among them, IP3 can stimulate endoplasmic reticulum, vacuoles, Golgi and other organelles to release Ca^2+^, thereby activating calcium signaling pathway [144,145,146]. Therefore, we propose a correlation between CaN-Crz1 signaling and the G protein-coupled receptor system (Figure 1). It was reported that glucose addition stimulates a rapid increase in free calcium level in yeast, thus activating the calcium signaling pathway [147,148]. Furthermore, Plc1p is essential for glucose-induced calcium increase. Studies suggest that Plc1p is activated by glucose firstly, and then lead to cleavaging PIP2 and generating IP3 for raising the calcium level in the cytosol [148]. However, in strains with a deletion in the *GPR1* or *GPA2* genes, the calcium influx induced by addition of high glucose was inhibited, which suggests the physiological process requires the Gpr1p/ Gpa2p receptor/G protein-coupled (GPCR) complex [149,150].

In *S. cerevisiae*, DNA microarray data indicated that a total of 150 genes responded to the alkaline pH environment, but the expression of many alkali-induced genes was inhibited in the CaN or Crz1 mutants, suggesting that calcium signaling is involved in the alkaline stress response [120]. The Rim101 signal transduction pathway is responsible for the adaptation of *C. albicans* to the alkaline environment [151]. Wang et al. [2] confirmed that *C. albicans* activated the calcium influx system in response to alkaline stress, and both Rim101 and Crz1 were involved in the activation of PHO89 promoter induced by alkaline stress, indicating that Rim101 and Crz1 signaling pathways had potential chelating effects in *C. albicans* response to alkaline stress. In addition, the interaction between CaN-Crz1 and heat shock proteins (Haps) is involved in response to different environmental stress conditions [152]. Hsp90 physically interacts with calcineurin and mediates echinocandin resistance in *C. albicans* [153]. In *A. fumigatus*, the MAPK, Hsp90, and calcineurin signaling pathways are linked and play a role in drug resistance and development [154]. These data show that cross talk between calcineurin-Crz1 and other signaling pathways is common but the detailed molecular mechanisms need to be investigated further.

## 4. Conclusions and Prosect

In response to complex environmental stimuli, fungi regulate multiple cellular metabolic processes by sensing intracellular Ca^2+^ concentration changes and then activating expressions of target genes. As an important transcription factor downstream of the calcium signaling pathway, Crz1 is highly conserved in fungi and plays a critical role in growth, development, tolerance to stress conditions and pathogenicity. Although our insight into Crz1 biological function has recently advanced with unprecedented speed, there are still some open research problems that urgently need to be addressed: (1) the specific molecular mechanism of Crz1 in transcriptional regulation of target genes in calcium homeostasis system still needs to be further elucidated, (2) It is necessary to further carry out genetic and biochemical analysis experiments combined with transcriptome sequencing technology to understand the metabolic pathway regulated by the transcription factor Crz1 in fungi, (3) new, environmentally safe, species-specific strategies for disease control, such as RNA interference (RNAi) technology, should be explored based on clarifying its regulatory mechanism of Crz1.

## Data Availability

Not applicable.

## References

[1] Greene V., Hong C., Schanne F., Bartelt D.C. (2002). Oxidative stress-induced calcium signalling in *Aspergillus nidulans*. Cell. Signal..

[2] Wang H., Liang Y., Zhang B., Zheng W., Xing L., Li M. (2011). Alkaline stress triggers an immediate calcium fluctuation in *Candida albicans* mediated by *Rim101p* and *Crz1p* transcription factors. FEMS Yeast Res..

[3] Courchesne W.E., Vlasek C., Klukovich R., Coffee S. (2011). Ethanol induces calcium influx via the Cch1-Mid1 transporter in *Saccharomyces cerevisiae*. Arch. Microbiol..

[4] Popa C.V., Dumitru I., Ruta L.L., Danet A.F., Farcasanu I.C. (2010). Exogenous oxidative stress induces Ca^2+^ release in the yeast *Saccharomyces cerevisiae*. FEBS J..

[5] Kraus P.R., Nichols C.B., Heitman J. (2005). Calcium- and calcineurin independent roles for calmodulin in *Cryptococcus neoformans* morphogenesis and high-temperature growth. Eukaryot. Cell..

[6] Bodvard K., Jörhov A., Blomberg A., Molin M., Käll M. (2013). The yeast transcription factor *Crz1* is activated by light in a Ca^2+^/calcineurin-dependent and PKA-independent manner. PLoS ONE.

[7] Edlind T., Smith L., Henry K., Katiyar S., Nickels J. (2002). Antifungal activity in *Saccharomyces cerevisiae* is modulated by calcium signalling. Mol. Microbiol..

[8] Bonilla M., Cunningham K.W. (2003). Mitogen-activated Protein kinase stimulation of Ca^2+^ signaling is required for survival of endoplasmic reticulum stress in yeast. Mol. Biol. Cell.

[9] Cui J., Kaandorp J.A., Sloot P.M., Lloyd C.M., Filatov M.V. (2009). Calcium homeostasis and signaling in yeast cells and cardiac myocytes. FEMS Yeast Res..

[10] Denis V., Cyert M.S. (2002). Internal Ca^2+^ release in yeast is triggered by hypertonic shock and mediated by a TRP channel homologue. J. Cell Biol..

[11] Stathopoulos-Gerontides A., Guo J.J., Cyert M.S. (1999). Yeast calcineurin regulates nuclear localization of the *Crz1p* transcription factor through dephosphorylation. Genes Dev..

[12] Thewes S. (2014). Calcineurin-Crz1 signaling in lower eukaryotes. Eukaryot. Cell.

[13] Rusnak F., Mertz P. (2000). Calcineurin: Form and function. Physiol. Rev..

[14] Cyert M.S. (2003). Calcineurin signaling in *Saccharomyces cerevisiae*: How yeast go crazy in response to stress. Biochem. Biophys. Res. Commun..

[15] Boeckeler K., Tischendorf G., Mutzel R., Weissenmayer B. (2006). Aberrant stalk development and breakdown of tip dominance in Dictyostelium cell lines with RNAi-silenced expression of calcineurin B. BMC Dev. Biol..

[16] Thewes S., Schubert S.K., Park K., Mutzel R. (2013). Stress and development in *Dictyostelium discoideum*: The involvement of the catalytic calcineurin A subunit. J. Basic Microbiol..

[17] Kumar R., Musiyenko A., Oldenburg A., Adams B., Barik S. (2004). Post-translational generation of constitutively active cores from larger phosphatases in the malaria parasite, *Plasmodium falciparum*: Implications for proteomics. BMC Mol. Biol..

[18] Moreno V.R., Agüero F., Tekiel V., Sánchez D.O. (2007). The calcineurin A homologue from *Trypanosoma cruzi* lacks two important regulatory domains. Acta Trop..

[19] Graef I.A., Chen F., Chen L., Kuo A., Crabtree G.R. (2001). Signals transduced by Ca^2+^/calcineurin and NFATc3/c4 pattern the developing vasculature. Cell.

[20] Graef I.A., Wang F., Charron F., Chen L., Neilson J., Tessier-Lavigne M., Crabtree G.R. (2003). Neurotrophins and netrins require calcineurin/NFAT signaling to stimulate outgrowth of embryonic axons. Cell.

[21] Nguyen T., Lindner R., Tedeschi A., Forsberg K., Green A., Wuttke A., Gaub P., Di Giovanni S. (2009). NFAT-3 is a transcriptional repressor of the growth-associated protein 43 during neuronal maturation. J. Biol. Chem..

[22] Beals C.R., Sheridan C.M., Turck C.W., Gardne P., Crabtree G.R. (1997). Nuclear export of NF-ATc enhanced by glycogen synthase kinase-3. Science.

[23] Fürstenau U., Schwaninger M., Blume R., Jendrusch E.M., Knepel W. (1999). Characterization of a novel calcium response element in the glucagon gene. J. Biol. Chem..

[24] Lawrence M.C., Bhatt H.S., Easom R.A. (2002). NFAT regulates insulin gene promoter activity in response to synergistic pathways induced by glucose and glucagon-like peptide-1. Diabetes.

[25] Liu S., Hou Y., Liu W., Lu C., Wang W., Sun S. (2015). Components of the calcium-calcineurin signaling pathway in fungal cells and their potential as antifungal targets. Eukaryot. Cell.

[26] Kmetzsch L., Staats C.C., Simon E., Fonseca F.L., Sobrino L., Rodrigues J., Leal A.L., Nimrichter L., Rodrigues M.L., Schrank A. (2010). The vacuolar Ca²^+^ exchanger *Vcx1* is involved in calcineurin-dependent Ca²^+^ tolerance and virulence in *Cryptococcus neoformans*. Eukaryot. Cell.

[27] Antebi A., Fink G.R. (1992). The yeast Ca^2+^-ATPase homologue, *PMR1*, is required for normal Golgi function and localizes in a novel Golgi-like distribution. Mol. Biol. Cell..

[28] Marchi V., Sorin A., Wei Y., Rao R. (1999). Induction of vacuolar Ca^2+^-ATPase and H^+^/Ca^2+^ exchange activity in yeast mutants lacking *Pmr1*, the Golgi Ca^2+^-ATPase. FEBS Lett..

[29] Cronin S.R., Rao R., Hampton R.Y. (2002). *Cod1p*/*Spf1p* is a P-type ATPase involved in ER function and Ca^2+^ homeostasis. J. Cell Biol..

[30] Muller E.M., Locke E.G., Cunningham K.W. (2001). Differential regulation of two Ca^2+^ influx systems by pheromone signaling in *Saccharomyces cerevisiae*. Genetics.

[31] Zhang Y., Zheng Q., Sun C., Song J., Gao L., Zhang S., Muñoz A., Read N.D., Lu L. (2016). Palmitoylation of the cysteine residue in the DHHC motif of a palmitoyl transferase mediates Ca^2+^ homeostasis in *Aspergillus*. PLoS Genet..

[32] Martin D.C., Kim H., Mackin N.A., Maldonado-Báez L., Evangelista C., Beaudry V., Dudgeon D., Erdman S., Cunningham K. (2011). New regulators of a high affinity Ca^2+^ Influx system revealed through a genome-wide screen in yeast. J. Biol. Chem..

[33] Ding X.H., Yu Q.L., Xu N., Wang Y.Z., Cheng X.X., Qian K.F., Zhao Q., Zhang B., Xing L.J., Li M.C. (2013). *Ecm7*, a regulator of HACS, functions in calcium homeostasis maintenance, oxidative stress response and hyphal development in *Candida albicans*. Fungal Genet. Biol..

[34] Harren K., Tudzynski B. (2013). *Cch1* and *Mid1* are functionally required for vegetative growth under low-calcium conditions in the phytopathogenic ascomycete *Botrytis cinerea*. Eukaryot. Cell.

[35] Yu Q., Wang H., Cheng X., Xu N., Ding X.H., Xing L.J., Li M.C. (2012). Roles of *Cch1* and *Mid1* in morphogenesis, oxidative stress response and virulence in *Candida albicans*. Mycopathologia.

[36] Xu D.Y., Cheng J.Q., Cao C.L., Wang L.T., Jiang L.H. (2015). Genetic interactions between *Rch1* and the high-affinity calcium influx system Cch1/Mid1/Ecm7 in the regulation of calcium homeostasis, drug tolerance, hyphal development and virulence in *Candida albicans*. FEMS Yeast Res..

[37] Wang Y.N., Wang J.J., Cheng J.Q., Xu D.Y., Jiang L.H. (2015). Genetic interactions between the Golgi Ca^2+^/H^+^ exchanger *Gdt1* and the plasma membrane calcium channel Cch1/Mid1 in the regulation of calcium homeostasis, stress response and virulence in *Candida albicans*. FEMS Yeast Res..

[38] Brand A., Shanks S., Duncan V.M.S., Yang M., Mackenzie K., Gow N.A.R. (2007). Hyphal orientation of *Candida albicans* is regulated by a calcium-dependent mechanism. Curr. Biol..

[39] Wang S., Cao J.L., Liu X., Hu H.Q., Shi J., Zhang S.Z., Keller N.P., Lu L. (2012). Putative calcium channels *CchA* and *MidA* play the important roles in conidiation, hyphal polarity and cell wall components in *Aspergillus nidulans*. PLoS ONE.

[40] Muller E.M., Mackin N.A., Erdman S.E., Cunningham K.W. (2003). *Fig1p* facilitates Ca^2+^ influx and cell fusion during mating of *Saccharomyces cerevisiae*. J. Biol. Chem..

[41] Cavinder B., Trail F. (2012). Role of *Fig1*, a component of the low-affinity calcium uptake system, in growth and sexual development of filamentous fungi. Eukaryot. Cell.

[42] Qian H., Chen Q., Zhang S., Lu L. (2018). The claudin family protein figa mediates Ca^2+^ homeostasis in response to extracellular stimuli in *Aspergillus nidulans* and *Aspergillus fumigatus*. Front. Microbiol..

[43] Brand A., Lee K., Veses V., Gow N.A. (2009). Calcium homeostasis is required for contact-dependent helical and sinusoidal tip growth in *Candida albicans* hyphae. Mol. Microbiol..

[44] Zhang S., Zheng H., Long N., Carbo N., Chen P., Aguilar P.S., Lu L. (2014). *FigA*, a putative homolog of low-affinity calcium system member *Fig1* in *Saccharomyces cerevisiae*, is involved in growth and asexual and sexual development in *Aspergillus nidulans*. Eukaryot. Cell.

[45] Morris Z., Sinha D., Poddar A., Morris B., Chen Q. (2019). Fission yeast TRP channel *Pkd2p* localizes to the cleavage furrow and regulates cell separation during cytokinesis. Mol. Biol. Cell..

[46] Wang H.C., Chen Q.Y., Zhang S.Z., Lu L. (2021). A transient receptor potential-like calcium ion channel in the filamentous fungus *Aspergillus nidulans*. J. Fungi.

[47] Hou C.C., Tian W., Kleist T., He K., Garcia V., Bai F.L., Hao Y.L., Luan S., Li L.G. (2014). DUF221 proteins are a family of osmosensitive calcium permeable cation channels conserved across eukaryotes. Cell Res..

[48] Jiang L., Pan H. (2018). Functions of *CaPhm7* in the regulation of ion homeostasis, drug tolerance, filamentation and virulence in *Candida albicans*. BMC Microbiol..

[49] Stempinski P.R., Goughenour K.D., du Plooy L.M., Alspaugh J.A., Olszewski M.A., Kozubowski L. (2022). The *Cryptococcus neoformans Flc1* homologue controls calcium homeostasis and confers fungal pathogenicity in the infected hosts. mBio.

[50] Martín J.F. (2022). Vacuolal and peroxisomal calcium ion transporters in yeasts and fungi: Key role in the translocation of intermediates in the biosynthesis of fungal metabolites. Genes.

[51] Zhou X.L., Batiza A.F., Loukin S.H., Palmer C., Kung C., Saimi Y. (2003). The transient receptor potential channel on the yeast vacuole is mechanosensitive. Proc. Natl. Acad. Sci. USA.

[52] Palmer C.P., Zhou X.L., Lin J., Loukin S.H., Kung C., Saimi Y. (2001). A TRP homologin *Saccharomyces cerevisiaeforms* an intracellular Ca^2+^-permeable channel in the yeast vacuolar membrane. Proc. Natl. Acad. Sci. USA.

[53] Rigamonti M., Groppi S., Belotti F., Ambrosini R., Filippi G., Martegani E., Tisi R. (2015). Hypotonic stress-induced calcium signaling in *Saccharomyces cerevisiae* involves TRP-like transporters on the endoplasmic reticulum membrane. Cell Calcium.

[54] Aiello D.P., Fu L., Miseta A., Sipos K., Bedwell D.M. (2004). The Ca^2+^ homeostasis defects in a *pgm2Δ* strain of *Saccharomyces cerevisiae* are caused by excessive vacuolar Ca^2+^ uptake mediated by the Ca^2+^-ATPase *Pmc1p*. J. Biol. Chem..

[55] Forster C., Kane P.M. (2000). Cytosolic Ca^2+^ homeostasis is a constitutive function of the V-ATPase in *Saccharomyces cerevisiae*. J. Biol. Chem..

[56] Pittman J.K. (2011). Vacuolar Ca^2+^ uptake. Cell Calcium..

[57] Cunningham K.W. (2011). Acidic calcium stores of *Saccharomyces cerevisiae*. Cell Calcium.

[58] Bowman B.J., Draskovic M., Freitag M., Bowma E.J. (2009). Structure and distribution of organelles and cellular location of calcium transporters in *Neurospora crassa*. Eukaryot. Cell.

[59] Miseta A., Kellermayer R., Aiello D.P., Fu L., Bedwell D.M. (1999). The vacuolar Ca^2+^/H^+^ exchanger *Vcx1p*/*Hum1p* tightly controls cytosolic Ca^2+^ levels in *S. cerevisiae*. FEBS Lett..

[60] Cunningham K.W., Fink G.R. (1996). Calcineurin inhibits *VCX1*-dependent H^+^/Ca^2+^ exchange and induces Ca^2+^ ATPases in *Saccharomyces cerevisiae*. Mol. Cell. Biol..

[61] Cai X., Lytton J. (2004). The cation/Ca^2+^ exchanger superfamily: Phylogenetic analysis and structural implications. Mol. Biol. Evol..

[62] Shigaki T., Barkla B.J., Miranda-Vergara M.C., Zhao J., Pantoja O., Hirschi K.D. (2005). Identification of a crucial histidine involved in metal transport activity in the *Arabidopsis* cation/H^+^ exchanger *CAX1*. J. Biol. Chem..

[63] Shigaki T., Rees I., Nakhleh L., Hirschi K.D. (2006). Identification of three distinct phylogenetic groups of CAX cation/proton antiporters. J. Mol. Evol..

[64] Pozos T.C., Sekler I., Cyert M.S. (1996). The product of *HUM1*, a novel yeast gene is required for vacuolal Ca^2+^/H^+^ exchange and is related to mammalian Na^+^/Ca^2+^ exchangers. Mol. Cell Biol..

[65] Durr G., Strayle J., Plemper R., Elbs S., Klee S.K., Catty P., Wolf D.H., Rudolph H.K. (1998). The medial-Golgi ion pump *Pmr1* supplies the yeast secretory pathway with Ca^2+^ and Mn^2+^ required for glycosylation, sorting, and endoplasmic reticulum-associated protein degradation. Mol. Biol. Cell..

[66] Rudolph H.K., Antebi A., Fink G.R., Buckley C.M., Dorman T.E., LeVitre J., Davidow L.S., Mao J.I., Moir D.T. (1989). The yeast secretory pathway is perturbed by mutations in *PMR1*, a member of a Ca^2+^-ATPase family. Cell.

[67] Bates S., MacCallum D.M., Bertram G., Munro C.A., Hughes H.B., Buurman E.T., Brown A.J.P., Odds F.C., Gow N.A.R. (2005). *Candida albicans Pmr1p*, a secretory pathway P-type Ca^2+^/Mn^2+^-ATPase, is required for glycosylation and virulence. J. Biol. Chem..

[68] Pinchai N., Juvvadi P.R., Fortwendel J.R., Perfect B.Z., Rogg L.E., Asfaw Y.G., Steinbach W.J. (2010). The *Aspergillus fumigatus* P-type Golgi apparatus Ca^2+^/Mn^2+^ ATPase *PmrA* is involved in cation homeostasis and cell wall integrity but is not essential for pathogenesis. Eukaryot. Cell.

[69] Soriani F.M., Martins V.P., Magnani T., Tudella V.G., Curti C., Uyemura S.A. (2005). A *PMR1*-like calcium ATPase of *Aspergillus fumigatus*: Cloning, identification and functional expression in *S. cerevisiae*. Yeast.

[70] Cunningham L.L. (2005). The use of calcium phosphate cements in the maxillofacial region. J. Long Term Eff. Med. Implant..

[71] Huang Y., Li Y.C., Li D.M., Bi Y., Prusky D.B., Dong Y.P., Wang T.L., Zhang M., Zhang X.M., Liu Y.X. (2020). Phospholipase C from *Alternaria alternata* is induced by physiochemical cues on the pear fruit surface that dictate infection structure differentiation and pathogenicity. Front. Microbiol..

[72] Carafoli E., Genazzani A., Guerini D. (1999). Calcium controls the transcription of its own transporters and channels in developing neurons. Biochem. Biophys. Res. Commun..

[73] Zhang M., Tanaka T., Ikura M. (1995). Calcium-induced conformational transition revealed by the solution structure of apo calmodulin. Nat. Struct. Mol. Biol..

[74] Chin D., Means A.R. (2000). Calmodulin: A prototypical calcium sensor. Trends Cell Biol..

[75] Juvvadi P.R., Arioka M., Nakajima H., Kitamoto K. (2001). Cloning and sequence analysis of *cnaA* gene encoding the catalytic subunit of calcineurin from *Aspergillus oryzae*. FEMS Microbiol Lett..

[76] Juvvadi P.R., Fortwendel J.R., Rogg L.E., Burns K.A., Randell S.H., Steinbach W.J. (2011). Localization and activity of the calcineurin catalytic and regulatory subunit complex at the septum is essential for hyphal elongation and proper septation in *Aspergillus fumigatus*. Mol. Microbiol..

[77] Juvvadi P.R., Gehrke C., Fortwendel J.R., Lamoth F., Soderblom E.J., Cook E.C., Hast M.A., Asfaw Y.G., Creamer T.P., Steinbach W.J. (2013). Phosphorylation of calcineurin at a novel serine-proline rich region orchestrates hyphal growth and virulence in *Aspergillus fumigatus*. PLoS Pathog..

[78] Griffith J.P., Kim J.L., Kim E.E., Sintchak M.D., Thomson J.A., Fitzgibbon M.J., Fleming M.A., Caron P.R., Hsiao K., Navia M.A. (1995). X-ray structure of calcineurin inhibited by the immunophilin-immunosuppre-ssant FKBP12-FK506 complex. Cell.

[79] Yang S.A., Klee C.B. (2000). Low affinity Ca^2+^-binding sites of calcineurin B mediate conformational changes in calcineurin A. Biochemistry.

[80] Sukumaran P., Conceicao V.N.D., Sun Y.Y., Ahamad N., Saraiva L.R., Selvaraj S., Singh B.B. (2021). Calcium signaling regulates autophagy and apoptosis. Cell.

[81] Sanglard D., Ischer F., Marchetti O., Entenza J., Bille J. (2003). Calcineurin A of *Candida albicans*: Involvement in antifungal tolerance, cell morphogenesis and virulence. Mol. Microbiol..

[82] Polizotto R.S. (2001). Calcineurin-dependent nuclear import of the transcription factor *Crz1p* requires *Nmd5p*. J. Cell Biol..

[83] Martin N., Bernard D. (2007). Calcium signaling and cellular senescence. Cell Calcium.

[84] Park H.S., Chow E.W.L., Fu C., Soderblom E.J., Moseley M.A., Heitman J., Cardenas M.E. (2016). Calcineurin targets involved in stress survival and fungal virulence. PLoS Pathog..

[85] Juvvadi P.R., Lee S.C., Heitman J., Steinbach W.J. (2017). Calcineurin in fungal virulence and drug resistance: Prospects for harnessing targeted inhibition of calcineurin for an antifungal therapeutic approach. Virulence.

[86] Yang Y.Y., Xie P.D., Yuan J., Liu Y.X., Zhang M., Li Y.C., Bi Y., Prusky D.B. (2022). The calcineurin-responsive transcription factor Crz1 is required for regulation of infection structure differentiation, calcium homeostasis and cell wall integrity in *Alternaria alternata*. Postharvest Biol. Technol..

[87] Jiang Q.Q., Mao R.Y., Li Y.C., Bi Y., Liu Y.X., Zhang M., Li R., Yang Y.Y., Prusky D.B. (2022). *AaCaM* is required for infection structure differentiation and secondary metabolites in pear fungal pathogen *Alternaria alternata*. J. Appl. Microbiol..

[88] Stathopoulos A.M., Cyert M.S. (1997). Calcineurin acts through the *CRZ1*/*TCN1*-encoded transcription factor to regulate gene expression in yeast. Genes Dev..

[89] Choi J., Kim Y., Kim S., Park J., Lee Y. (2009). *Mo**CRZ1*, a gene encoding a calcineurin-responsive transcription factor, regulates fungal growth and pathogenicity of *Magnaporthe oryzae*. Fungal Genet. Biol..

[90] Schumacher J., De Larrinoa I.F., Tudzynski B. (2008). Calcineurin-responsive zinc finger transcription factor *CRZ1* of *Botrytis cinerea* is required for growth, development, and full virulence on bean plants. Eukaryot. Cell.

[91] Soriani F.M., Malavazi I., Ferreira M.E., Savoldi M., Goldman M.H. (2008). Functional characterization of the *Aspergillus fumigatus*
*CRZ1* homologue, *CrzA*. Mol. Microbiol..

[92] Matheos D.P., Kingsbury T.J., Ahsan U.S., Cunningham K.W. (1997). *Tcn1p*/*Crz1p*, a calcineurin-dependent transcription factor that differentially regulates gene expression in *Saccharomyce scerevisiae*. Genes Dev..

[93] Yoshimoto H., Saltsman K., Gasch A.P., Li H.X., Ogawa N., Botstein D., Cyert M.S. (2002). Genomewide analysis of gene expression regulated by the calcineurin/*Crz1p* signaling pathway in *Saccharomyces cerevisiae*. J. Biol. Chem..

[94] Karababa M., Valentino E., Pardini G., Coste A.T., Bille J., Sanglard D. (2006). CRZ1, a target of the calcineurin pathway in *Candida albicans*. Mol. Microbiol..

[95] Boustany L.M., Cyert M.S. (2002). Calcineurin-dependent regulation of *Crz1p* nuclear export requires *Msn5p* and a conserved calcineurin docking site. Genes Dev..

[96] Kafadar K.A., Zhu H., Snyder M., Cyert M.S. (2003). Negative regulation of calcineurin signaling by *Hrr25p*, a yeast homolog ofcasein kinase I. Genes Dev..

[97] Kaffman A., Rank N.M., O’Neill E.M., Huang S., O’Shea E.K. (1998). The receptor *Msn5* exports the phosphorylated transcription factor *Pho4* out of the nucleus. Nature.

[98] Zhao K.L., Liu Z.J., Li M.X., Hu Y.Y., Yang L., Song X., Qin Y.Q. (2021). Drafting *Penicillium oxalicum* calcineurin-*CrzA* pathway by combining the analysis of phenotype, transcriptome, and endogenous protein-protein interactions. Fungal Genet Biol..

[99] Huang H.Y., Hopper A.K. (2015). In vivo biochemical analyses reveal distinct roles of *β*-importins and *eEF1A* in tRNA subcellular traffic. Genes Dev..

[100] Chen L., Tong Q., Zhang C., Ding K.J. (2018). The transcription factor *FgCrz1A* is essential for fungal development, virulence, deoxynivalenol biosynthesis and stress responses in *Fusarium graminearum*. Curr. Genet..

[101] Hagiwara D., Kondo A., Abe F.K. (2008). Functional analysis of C2H2 zinc finger transcription factor *CrzA* involved in calcium signaling in *Aspergillus nidulans*. Curr. Genet..

[102] Zhang T., Xu Q., Sun X., Li H. (2013). The calcineurin-responsive transcription factor *Crz1* is required for conidation, full virulence and DMI resistance in *Penicillium digitatum*. Microbiol. Res..

[103] Zhang H.F., Zhao Q., Liu K., Zhang Z., Wang Y., Zheng X. (2009). *Mg**CRZ1*, a transcription factor of *Magnaporthe grisea*, controls growth, development and is involved in full virulence. FEMS Microbiol. Lett..

[104] Xiong D., Wang Y., Tang C., Fang Y., Zou J., Tian C. (2015). *Vd**Crz1* is involved in microsclerotia formation and required for full virulence in *Verticillium dahliae*. Fungal Genet Biol..

[105] Zhang J., Silao F.G., Bigol U.G., Bungay A.A., Nicolas M.G., Heitman J., Chen Y.L. (2012). Calcineurin is required for pseudohyphal growth, virulence, and drug resistance in *Candida lusitaniae*. PLoS ONE.

[106] Chen Y.L., Konieczka J.H., Springer D.J., Bowen S.E., Zhang J., Silao F.G., Bungay A.A., Bigol U.G., Nicolas M.G., Abraham S.N. (2012). Convergent evolution of calcineurin pathway roles in thermotolerance and virulence in *Candida glabrata*. G3 Genes Genomes Genet..

[107] He F., Zhang X., Mafurah J.J., Zhang M., Qian G., Wang R., Safdar A., Yang X., Liu F., Dou D. (2016). The transcription factor *VpCRZ1* is required for fruiting body formation and pathogenicity in *Valsa pyri*. Microb. Pathog..

[108] Gao L., Song Y., Cao J., Wang S., Wei H., Jiang H.C., Lu L. (2011). Osmotic stabilizer-coupled suppression of NDR defects is dependent on the calcium-calcineurin signaling cascade in *Aspergillus nidulans*. Cell Signal..

[109] Onyewu C., Wormley F.L., Perfect J.R., Heitman J. (2004). The calcineurin target, *Crz1*, functions in azole tolerance but is not required for virulence of *Candida albicans*. Infect. Immun..

[110] Miyazaki T., Yamauchi S., Inamine T., Nagayoshi Y., Saijo T., Izumikawa K., Seki M., Kakeya H., Yamamoto Y., Yanagihara K. (2010). Roles of calcineurin and *Crz1* in antifungal susceptibility and virulence of *Candida glabrata*. Antimicrob. Agents Chemother..

[111] Hernandez-Lopez M.J., Panadero J., Prieto J.A., Randez-Gil F. (2006). Regulation of salt tolerance by *Torulaspora delbrueckii* calcineurin target *Crz1p*. Eukaryot. Cell.

[112] Spielvogel A., Findon H., Arst H., Lidia A.B., Patricia H.O., Stahl U., Meyer V., Espeso E.A. (2008). Two zinc finger transcription factors, *CrzA* and *SltA*, are involved in cation homoeostasis and detoxification in *Aspergillus nidulans*. Biochem. J..

[113] Luna-Tapia A., DeJarnette C., Sansevere E., Reitler P., Butts A., Hevener K.E., Palmer G.E. (2019). The vacuolar Ca^2+^-ATPase pump *Pmc1p* is required for *Candida albicans* pathogenesis. mSphere.

[114] Cunningham K.W., Fink G.R. (1994). Calcineurin-dependent growth control in *Saccharomyces cerevisiae* mutants lacking *PMC1*, a homolog of plasma membrane Ca^2+^ ATPases. J. Cell Biol..

[115] Tsuzi D., Maeta K., Takatsume Y., Izawa S., Inoue Y. (2004). Regulation of the yeast phospholipid hydroperoxide glutathione peroxidase GPX2 by oxidative stress is mediated by *YAP1* and *SKN7*. FEBS Lett..

[116] Tsuzi D., Maeta K., Takatsume Y., Izawa S., Inoue Y. (2004). Distinct regulatory mechanism of yeast GPX2 encoding phospholipid hydroperoxide glutathione peroxidase by oxidative stress and a calcineurin/Crz1-mediated Ca^2+^ signaling pathway. FEBS Lett..

[117] Williams K., Cyert M.S. (2001). The eukaryotic response regulator *SKN7* regulates calcineurin signalling through stabilization of *Crz1p*. EMBO J..

[118] Chen X., Liu Y.Z., Nemat O., Xia Y.X., Cao Y.Q. (2017). The regulatory role of the transcription factor *Crz1* in stress tolerance, pathogenicity, and its target gene expression in *Metarhizium acridum*. Appl. Microbiol. Bio..

[119] Viladevall L., Serrano R., Ruiz A., Domenech G., Giraldo J., Barceló A., Ariño J. (2004). Characterization of the calcium-mediated response to alkaline stress in *Saccharomyces cerevisiae*. J. Biol. Chem..

[120] Serrano R., Ruiz A., Bernal D., Chambers J.R., Ariño J. (2002). The transcriptional response to alkaline pH in *Saccharomyces cerevisiae*: Evidence for calcium-mediated signalling. Mol. Microbiol..

[121] Aboobakar E.F., Wang X.Y., Heitman J., Kozubowski L. (2011). The C2 domain protein *Cts1* functions in the calcineurin signaling circuit during high-temperature stress responses in *Cryptococcus neoformans*. Eukaryot. Cell.

[122] Araki Y., Hong W., Kitagaki H., Akao T., Takagi H., Shimoi H. (2009). Ethanol stress stimulates the Ca^2+^-mediated calcineurin/*Crz1* pathway in *Saccharomyces cerevisiae*. J. Biosci Bioeng..

[123] Moranova Z., Virtudazo E., Hricova K., Ohkusu M., Kawamoto S., Husickova V., Raclavsky V. (2013). The CRZ1/SP1-like gene links survival under limited aeration, cell integrity and biofilm formation in the pathogenic yeast *Cryptococcus neoformans*. Biomed. Pap..

[124] Dinamarco T.M., Freitas F.Z., Almeida R.S., Brown N.A., Reis T.F.d., Ramalho L.N.Z., Savoldi M., Goldman M.H.S., Bertolini M.C., Goldman G.H. (2012). Functional characterization of an *Aspergillus fumigatus* calcium transporter (*PmcA*) that is essential for fungal infection. PLoS ONE.

[125] Martins-Santana L., de Paula R.G., Gomes Silva A., Christian Borges Lopes D., do Nascimento Silva R., Silva-Rocha R. (2019). CRZ1 regulator and calcium cooperatively modulate holocellulases gene expression in *Trichoderma reesei QM6a*. Genet. Mol. Biol..

[126] Haro R., Garciadeblas B., Rodriguez-Navarro A. (1991). A novel P-type ATPase from yeast involved in sodium transport. Genet. Mol. Biol..

[127] Adler A., Park Y.D., Larsen P., Nagarajan V., Wollenberg K., Qiu J., Myers T.G., Williamson P.R. (2011). A novel specificity protein 1 (SP1)-like gene regulating protein kinase C-1 (Pkc1) dependent cell wall integrity and virulence factors in *Cryptococcus neoformans*. J. Biol. Chem..

[128] Lev S., Desmarini D., Chayakulkeeree M., Sorrell T.C., Djordjevic J.T. (2012). The *Crz1*/*Sp1* transcription factor of *Cryptococcus neoformans* is activated by calcineurin and regulates cell wall integrity. PLoS ONE.

[129] Chen Y.L., Brand A., Morrison E.L., Silao F.G., Bigol U.G., Malbas F.F., Nett J.E., Andes D.R. (2011). Calcineurin controls drug tolerance, hyphal growth, and virulence in *Candida dubliniensis*. Eukaryot. Cell.

[130] Fuchs B.B., Mylonakis E. (2009). Our paths might cross: The role of the fungal cell wall integrity pathway in stress response and cross talk with other stress response pathways. Eukaryot. Cell.

[131] Levin D.E. (2011). Regulation of cell wall biogenesis in *Saccharomyces cerevisiae*: The cell wall integrity signaling pathway. Genetics.

[132] Zhao C., Jung U.S., Garrett-Engele P., Roe T., Cyert M.S., Levin D.E. (1998). Temperature-induced expression of yeast *FKS2* is under the dual control of protein kinase C and calcineurin. Mol. Cell Biol..

[133] Wang X., Sheff M.A., Simpson D.M., Elion E.A. (2011). *Ste11p* MEKK signals through HOG, mating, calcineurin and PKC pathways to regulate the *FKS2* gene. BMC Mol. Biol..

[134] Brewster J., Valoir T.D., Dwyer N., Winter E., Gustin M.C. (1993). An osmosensing signal transduction pathway in yeast. Science.

[135] Maeda T., Takekawa M., Saito H. (1995). Activation of yeast *PBS2* MAPKK by MAPKKKs or by binding of an SH3-containing osmosensor. Science.

[136] Posas F., Wurgler-Murphy S.M., Maeda T., Witten E.A., Thai T.C., Saito H. (1996). Yeast HOG1 MAP kinase cascade is regulated by a multistep phosphorelay mechanism in the SLN1-YPD1-SSK1 “two-component” osmosensor. Cell.

[137] Posas F., Saito H. (1997). Osmotic activation of the HOG MAPK pathway via *Ste11p* MAPKKK: Scaffold role of *Pbs2p* MAPKK. Science.

[138] Philips J., Herskowitz I. (1997). Osmotic balance regulates cell fusion during mating in *Saccharomyces cerevisiae*. J. Cell Biol..

[139] Shitamukai A., Hirata D., Sonobe S., Miyakawa T. (2004). Evidence for antagonistic regulation of cell growth by the calcineurin and high osmolarity glycerol pathways in *Saccharomyces cerevisiae*. J. Biol. Chem..

[140] Kafadar K.A., Cyert M.S. (2004). Integratiom of stress responses: Modulation of calcineurin signaling in *Saccharomyces cerevisiae* by protein kinase A. Eukaryot. Cell..

[141] Hamm H.E. (1998). The many faces of G protein signaling. J. Biol. Chem..

[142] Li L., Wright S.J., Krystofova S., Park G., Borkovich K.A. (2007). Heterotrimeric G protein signaling in filamen-tous fungi. Annu. Rev. Microbiol..

[143] Berridge M.J. (1993). Inositol trisphosphate and calcium signalling. Nature.

[144] Anjago W.M., Zhou T., Zhang H. (2018). Regulatory network of genes associated with stimuli sensing, signal transduction and physiological transformation of appressorium in *Magnaporthe oryzae*. Mycology.

[145] Singh A., Bhatnagar N., Pandey A. (2015). Plant phospholipase C family: Regulation and functional role in lipid signaling. Cell Calcium..

[146] Avishek R., Ajeet K., Darshana B., Tamuli R. (2020). Calcium signaling is involved in diverse cellular processes in fungi. Mycology.

[147] Groppi S., Belotti F., Brandão R.L., Martegani E., Tisi R. (2011). Glucose-induced calcium influx in budding yeast involves a novel calcium transport system and can activate calcineurin. Cell Calcium.

[148] Tisi R., Belotti F., Wera S., Winderickx J., Thevelein J.M., Martegani E. (2004). Evidence for inositol triphosphate as a second messenger for glucose-induced calcium signalling in budding yeast. Curr. Genet..

[149] Tisi R., Baldassa S., Belotti F., Martegani E. (2002). Phospholipase C is required for glucose-induced calcium influx in budding yeast. FEBS Lett..

[150] Coccetti P., Tisi R., Martegani E., Teixeira L.S., Brandão R.L., Thevelein J.M. (1998). The *PLC1* encoded phospholipase C in the yeast *Saccharomyces cerevisiae* is essential for glucose-induced phosphatidylinositol turnover and activation of plasma membrane H^+^-ATPase. BBA Mol. Cell Res..

[151] Bensen E.S., Martin S.J., Li M., Berman J., Davis D.A. (2004). Transcriptional profiling in *Candida albicans* reveals new adaptive responses to extracellular pH and functions for *Rim101p*. Mol. Microbiol..

[152] Roy A., Tamuli R. (2022). Heat shock proteins and the calcineurin-Crz1 signaling regulate stress responses in fungi. Arch. Microbiol..

[153] Singh S.D., Robbins N., Zaas A.K., Schell W.A., Perfect J.R., Cowen L.E. (2009). Hsp90 governs echinocandin resistance in the pathogenic yeast *Candida albicans* via calcineurin. PLoS Pathog..

[154] O’Meara T.R., Cowen L.E. (2014). Hsp90-dependent regulatory circuitry controlling temperature-dependent fungal development and virulence. Cell Microbiol..

